# Spin-coated $\hbox {Cu}_2\hbox {ZnSnS}_{4}$ solar cells: A study on the transformation from ink to film

**DOI:** 10.1038/s41598-020-77592-z

**Published:** 2020-11-27

**Authors:** Sara Engberg, Filipe Martinho, Mungunshagai Gansukh, Alexander Protti, Rainer Küngas, Eugen Stamate, Ole Hansen, Stela Canulescu, Jørgen Schou

**Affiliations:** 1 DTU Fotonik, Technical University of Denmark, 4000 Roskilde, Denmark; 2 Haldor Topsøe, 2800 Kgs. Lyngby, Denmark; 3 DTU Nanolab, Technical University of Denmark, 2800 Kgs. Lyngby, Denmark

**Keywords:** Materials for devices, Inorganic chemistry, Materials science, Solar cells

## Abstract

In this paper, we study the DMSO/thiourea/chloride salt system for synthesis of pure-sulfide $\hbox {Cu}_2\hbox {ZnSnS}_{4}$ (CZTS) thin-film solar cells under ambient conditions. We map out the ink constituents and determine the effect of mixing time and filtering. The thermal behavior of the ink is analyzed, and we find that more than 90% of the solvent has evaporated at $250\,^{\circ }\hbox {C}$. However, chloride and sulfoxide species are released continually until $500\,^{\circ }\hbox {C}$, suggesting the advantage of a higher pre-annealing temperature, which is also commonly observed in the spin-coating routines in literature. Another advantage of a higher pre-annealing temperature is that the worm-like pattern in the spin-coated film can be avoided. We hypothesize that this pattern forms as a result of hydrodynamics within the film as it dries, and it causes micro-inhomogeneities in film morphology. Devices were completed in order to finally evaluate the effect of varying thermal exposure during pre-annealing. Contrary to the previous observations, a lower pre-annealing temperature of $250\,^{\circ }\hbox {C}$ results in the best device efficiency of 4.65%, which to the best of our knowledge is the highest efficiency obtained for a pure-sulfide kesterite made with DMSO. Lower thermal exposure during pre-annealing results in larger grains and a thicker $\hbox {MoS}_2$ layer at the CZTS/Mo interface. Devices completed at higher pre-annealing temperatures display the existence of either a Cu-S secondary phase or an incomplete sulfurization with smaller grains and a fine-grain layer at the back interface.

## Introduction

Our renewable energy future depends on the continuous development of the photovoltaic (PV) technology. Earth-abundant raw materials and an inexpensive production method are required for terawatt level PV deployment. One key compound in the search for the next-generation absorber is the kesterite ($\hbox {Cu}_{2}\hbox {ZnSn(S}_{x}\hbox {Se}_{1-x})_{4}$ or CZTSSe): As a p-type semiconductor with a tunable band gap and high absorption coefficient^1^, it is considered the potential successor of $\hbox {Cu(In,Ga)Se}_{2}$ (CIGS) in the field of thin-film PV^2^. In particular, the pure-sulfide $\hbox {Cu}_2\hbox {ZnSnS}_{4}$ (CZTS) contains neither toxic (Cd or Se) nor rare (In and Ga) elements. Additionally, it adapts a similar device architecture and thus comparable manufacturing facilities as the commercially available CIGS.

Of the fabrication methods available to synthesize CZTSSe, solution-processing is interesting from an economical perspective^3^. Non-vacuum methods offer a lower environmental impact due to lower electricity consumption in the manufacturing stage^4^, and lower capital expenditure (CAPEX) for establishing production lines^5^. A recent review by Todorov et al. presents the current state-of-the-art of five different solution-based approaches: (1) Hydrazine-based, (2) Aprotic molecular inks, (3) Protic solvents and the sol–gel method, (4) Nanoparticle inks, and (5) Electrodeposition, as well as the strengths, weaknesses, opportunities, and threats (SWOT) of the different solution-based synthetis routes^2^. The hydrazine-based and the aprotic molecular inks are solutions, i.e. single-phase homogeneous liquids, as opposed to heterogeneous mixtures, as is the case for slurries, suspensions, or colloidal dispersions of nanoparticles. The CZTSSe record power conversion efficiency (PCE) of 12.6% was achieved by using the solvent hydrazine^6^, however, the dangerous nature of the solvent would require strict safety and environmental processing conditions in industrial settings. The other approaches contain more benign chemistry, and in general, the challenges now lie in removing a carbon-rich layer or avoiding a multilayer morphology^2^.

In recent years, efficiencies exceeding 10% for solution-processed CZTSSe have been demonstrated using aprotic molecular inks^7^. This was achieved using the solvent dimethyl sulfoxide (DMSO) and salts of thiourea (TU) and metal chlorides, and without any additional elements—a method first described by Ki and Hillhouse^8^. However, the DMSO route for the pure-sulfide counterpart remains rather unexplored, with an efficiency of 3.05% for the undoped kesterite^9^. For vacuum-made devices, CZTSSe and CZTS are much closer in performance level with efficiencies of 12.6% and 11.0%, respectively^10,11^, which suggests that the DMSO-deposited pure-sulfide CZTS has significant potential for improvement. The interest in the high band gap kesterite is also rekindled from the recent development in tandem devices^12,13^. For tandem applications, a higher band gap of 1.5–1.7 eV is favorable, and thus the sulfide CZTS and its alloys are considered.

The DMSO route is attractive since it is binder-free, offers effective metal complexing, and contains a high concentration of chalcogen in the ink (as part of both thiourea and DMSO), which could improve spatial uniformity^9^. The recent advances in the DMSO-processed sulfo-selenide kesterite originate from using cations with the correct oxidation number^7^, incorporating lithium^5,14,15^, adjusting the thermal exposure during pre-annealing^5^, or changing the ink properties^16–19^. An overview of ink formulations, pre-annealing conditions, and device performances is presented in Table 1, for both CZTS and CZTSSe.

**Table 1:** Performance, ink properties and pre-annealing conditions for published spin-coated aprotic molecular inks with DMSO.

	Performance		Pre-annealing conditions			Ink properties				References
	Additives	PCE (%)	Temp. ($^{\circ }$C)	Time (min)	Atm.	Mixing time	Salts			
CZTS	–/Na	3.05/4.53	320	–	Air		$\hbox {CuCl}_{2}$	$\hbox {ZnCl}_2$	$\hbox {SnCl}_{2}$	^9^
	–	1.9	350	1	Air	Hours	$\hbox {Cu(OAc)}_{2}\times \hbox {H}_{2}\hbox {O}$	$\hbox {ZnCl}_{2}$	$\hbox {SnCl}_{2}\times 2\hbox {H}_{2}\hbox {O}$	^20^
CZTSSe	–	13.6^a,b^	420	2	$\hbox {N}_2$		CuCl	$\hbox {Zn(OAc)}_{2}$	$\hbox {SnCl}_{4}$	^21^
	–/Li	8.7/11.8^a^	540	2	$\hbox {N}_2$	Overnight	$\hbox {Cu(OAc)}_{2}\times \hbox{H}_{2}\hbox {O}$	$\hbox {ZnCl}_{2}$	$\hbox {SnCl}_{2}\times 2\hbox {H}_{2}\hbox {O}$	^14^
	–/Li	<6/11.5	320	1	Air		$\hbox {CuCl}_{2}\times 2\hbox {H}_{2}\hbox {O}$	$\hbox {ZnCl}_{2}$	$\hbox {SnCl}_{2}\times 2\hbox {H}_{2}\hbox {O}$	^15^
	–/Li	9.1/10.7^a^	500	1.5	$\hbox {N}_2$	Overnight	$\hbox {Cu(OAc)}_{2}\times \hbox{H}_{2}\hbox {O}$	$\hbox {ZnCl}_{2}$	$\hbox {SnCl}_{2}\times 2\hbox {H}_{2}\hbox {O}$	^5^
	–	10.0	420	1.5	$\hbox {N}_2$	Overnight	CuCl	$\hbox {Zn(OAc)}_{2}\times 2\hbox {H}_{2}\hbox {O}$	$\hbox {SnCl}_{4}\times 5\hbox {H}_{2}\hbox {O}$	^7^
	$\hbox {H}_2$O	9.3	350	Quick	$\hbox {N}_2$	3 h	$\hbox {Cu(OAc)}_{2}\times \hbox{H}_{2}\hbox {O}$	$\hbox {Zn(OAc)}_{2}\times 2\hbox {H}_{2}\hbox {O}$	$\hbox {SnCl}_{2}$	^16,17^
	DMF	8.6	300	2	Air	3 h	$\hbox {Cu(NO}_{3})_{2}\times 3\hbox {H}_{2}\hbox {O}$	$\hbox {Zn(OAc)}_{2}\times 2\hbox {H}_{2}\hbox {O}$	$\hbox {SnCl}_{2}\times 2\hbox {H}_{2}\hbox {O}$	^18^
	$\hbox {H}_3\hbox {BO}_3$	7.3	540	5	Ar	24 h	$\hbox {Cu(OAc)}_{2}$	$\hbox {ZnCl}_{2}$	$\hbox {SnCl}_{2}\times 2\hbox {H}_{2}\hbox {O}$	^19^

^a^Filtered ($0.8\,\upmu \hbox {m}$ PTFE^21^, $2\,\upmu \hbox {m}$ PTFE^14^, and $0.2\,\upmu \hbox {m}$ PTFE^5^).

^b^Active area efficiency.

An important part of fabricating an efficient solution-processed solar cell is transforming the liquid ink into a solid film, and understanding the factors contributing to the final stoichiometry, phase, and morphology of the absorber is crucial. Today, few studies exist on the formation mechanism of the kesterite via the aprotic solvent route. Clark et al. recently described the complexation chemistry in similar systems, providing a new understanding of the chalcogenide molecular inks^22^. The coordination complexes that form in a molecular ink typically consist of a central metallic atom or ion surrounded by an array of covalently bound molecules or ions, referred to as ligands or complexing agents. It is important that the ink is mixed sufficiently to ensure that the salts have dissolved and complexes have formed. In the next step, filtering the ink before deposition is commonly done, probably because it results in a more uniform film, as agglomerates are avoided. Upon film deposition, several processes can occur during the drying process: The solvent will evaporate and the complexes will thermally decompose. Depending on the pre-annealing temperature, the formation of some phases can already occur. In addition, there can be hydrodynamic processes related to drying the wet film, or the spin-coating procedure itself can introduce re-dissolution of the film when a new layer is deposited—perhaps preferentially re-dissolving certain elements and thus affecting the composition. It is important to study the transformation from ink to film, to understand and tune the processes occurring.

**Figure 1:**
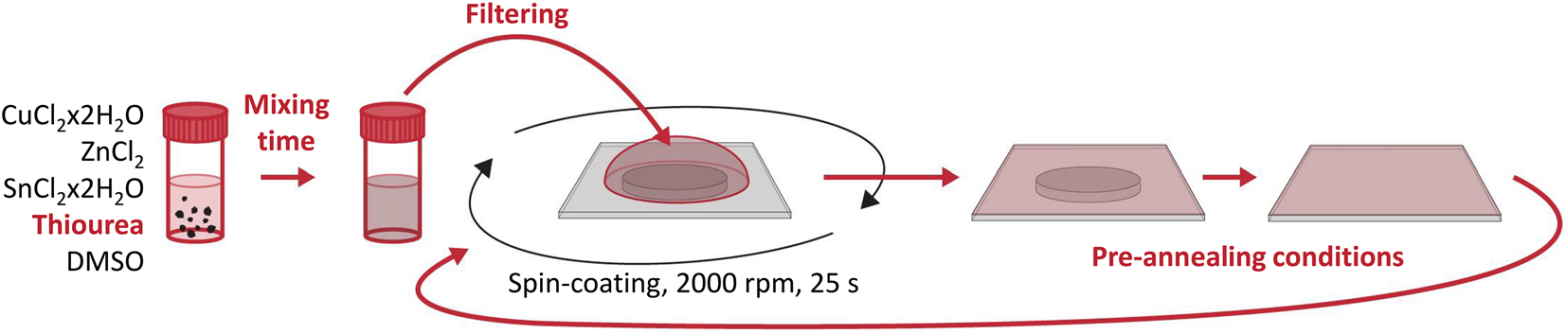
Route for formulating an aprotic molecular ink and spin-coating consecutive layers. The steps marked in bold and red, are investigated in this paper, i.e. the effect of thiourea (TU) concentration, mixing time, filtering, and pre-annealing conditions.

In this work, we explore a simple approach based on the utilization of hydrated chloride salts for the synthesis of CZTS thin films. By doing so, we avoid the use of liquid or air-sensitive salts, and this enables materials processing under ambient conditions. The ink formulation and spin-coating procedure studied in this work is depicted in Fig. 1. In the first part of the paper, we determine a suitable formulation and study the effects of thiourea concentration, mixing time, filtering, and heating on the properties of the aprotic molecular ink. In the second part, we study at the device level how the processing of the ink influences the device performance, and a study on thermal exposure during the pre-annealing step is carried out. Finally, we summarize our findings and discuss the mechanism of formation.

According to Todorov et al., the main challenge of the aprotic molecular ink approach lies in controlling the thermal conditions during annealing to ensure removal of C and N species while forming dense grains^2^. By optimizing the thermal exposure during pre-annealing, we achieve a morphology consisting of large and densely packed grains of CZTS. We find that the CZTS grain size, the degree of sulfurization of the back Mo substrate into $\hbox {MoS}_2$, and formation of a multilayered structure vary with thermal exposure during pre-annealing. We observe that the highest performing solar cells are formed by low thermal exposure routes, contradicting the most prevalent methods used in the field^5,7,14^.

## Results

### The aprotic molecular ink

**Figure 2:**
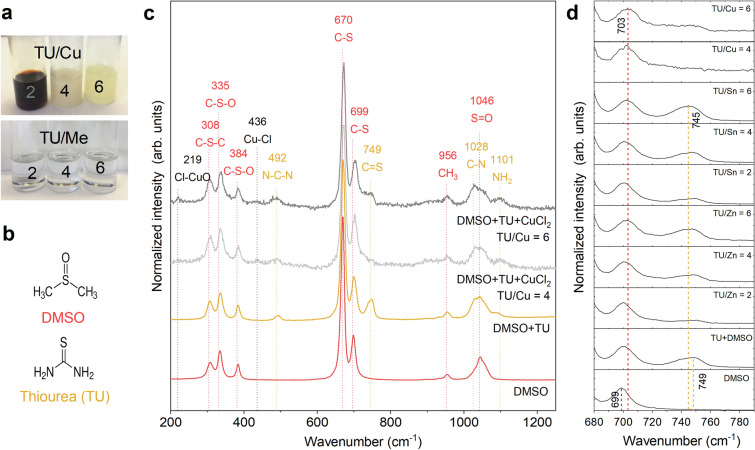
Investigation of the aprotic molecular ink. (**a**) Images of inks with different thiourea to copper (TU/Cu) and thiourea to metal (TU/Me) concentrations. (**b**) The molecular structure of DMSO in red and thiourea in yellow. (**c**) Raman spectra of liquid inks containing DMSO, DMSO and TU, as well as mixtures with DMSO, TU and TU/Cu ratios of 4 and 6. Major Raman modes are tentatively assigned to species, where red corresponds to DMSO, yellow to TU, and black to Cu. (**d**) Raman spectra of the DMSO C-S and the thiourea C=S stretching modes for inks consisting of $\hbox {DMSO}+\hbox {TU}+\hbox {CuCl}_2$, $\hbox {DMSO}+\hbox {TU}+\hbox {ZnCl}_2$, or $\hbox {DMSO}+\hbox {TU}+\hbox {SnCl}_2$ with different concentrations of TU.

The ink is prepared by mixing all salts with DMSO, and stirring it until a transparent liquid is achieved. During this process, it is essential that the cations adopt the correct oxidation state. The salts consist of Cu(II), Zn(II), and Sn(II), while CZTS requires Cu(I), Zn(II), and Sn(IV). The necessary redox reaction can only occur when all salts are mixed simultaneously, as the reduction of Cu(II) happens concurrently with the oxidation of Sn(II). Inks of simultaneously mixed salts are illustrated in Fig. 2a, where transparent solutions are obtained for mixtures containing different concentrations of thiourea (TU) to metals (Me), i.e. the TU/Me ratio, or different concentrations of TU to copper, i.e. the TU/Cu ratio. The Zn and Sn salts dissolve well in DMSO and thiourea (see Supplementary Fig. S1 online), however, the Cu inks become blackish and yellowish depending on the TU/Cu concentration (Fig. 2a). Similar mixtures have been investigated by Collard et al. and Xin et al. who found that the black and yellow colours stem from the existence of Cu(II)S nanoparticles and $\hbox {Cu(II)Cl}_2$ or $\hbox {Cu(II)DMSO}_2\hbox {Cl}_2$, respectively^14,26^. We also note that when preparing each cation solution separately, and subsequently combining them, the ink becomes yellow (not shown). This suggests that Cu(II) has not been fully reduced to Cu(I), possibly due to a higher stability of $\hbox {Cu(II)Cl}_2$ or $\hbox {Cu(II)DMSO}_2\hbox {Cl}_2$. The redox reaction could perhaps leave the system in a charge imbalance state, however, whether these charges affect the ink and/or deposition of it, is uncertain.

#### Complex formation

Metal complex formation was investigated by Raman spectroscopy for one metal at a time, using a similar methodology as Clark and coworkers^22^. Raman spectroscopy has proven a valuable tool for the characterization of inks and solids, and it can be used to identify broken covalent bonds in molecules, i.e. whether and how a molecule is bonded to a metal cation. This study aimed to ensure that the complexes were fully coordinated and to determine a suitable formulation for further study.

Figure 2 and Supplementary Fig. S2 show the Raman spectra of inks containing DMSO, DMSO and thiourea (TU), as well as mixtures of DMSO, thiourea, and one metal salt at different thiourea concentrations. The assignment of the different vibrational bands is performed in relation to literature references and enables us to infer the coordination of the metal cations in DMSO/thiourea solutions. The Raman spectrum of DMSO (red line, Fig. 2c) consists of several peaks between 308 and $1046\,\hbox {cm}^{-1}$, as listed in Table 2. These peaks are assigned to C–S–C bending, C–S–O bending, C–S stretching, $\hbox {CH}_3$ rocking, and S=O stretching^23^. In the case of a DMSO and TU solution (yellow line, Fig. 2c), additional Raman peaks appear, which can be assigned to thiourea; namely N–C–N bending, C=S stretching, C–N stretching, and $\hbox {NH}_2$ rocking^24^.

**Table 2:** Active Raman modes and tentatively assigned species^23–27^.

Species	Raman mode ($\hbox {cm}^{-1}$)	Chemical	Assigned mode
C–S–C	308	DMSO	Bending
C–S–O	335	DMSO	Asymmetric bending
C–S–O	384	DMSO	Symmetric bending
C–S	670	DMSO	Symmetric stretch
C–S	699	DMSO	Antisymmetric stretch
$\hbox {CH}_3$	956	DMSO	Rocking
S=O	1046	DMSO	Stretch
N–C–N	492	TU	Bending
C=S	749	TU	Stretch
C–N	1028	TU	Stretch
$\hbox {NH}_2$	1101	TU	Rocking
Cl–CuO	219	$\hbox {CuCl}_2\times 2\hbox {H}_2\hbox {O}$	Bending
Cu–Cl	436	$\hbox {CuCl}_2\times 2\hbox {H}_2\hbox {O}$	Symmetric stretching

Once $\hbox {CuCl}_{2}\times 2\hbox {H}_{2}\hbox {O}$ is added to the DMSO/thiourea solution, we find that the intensity of the Raman band at $749\,\hbox {cm}^{-1}$ (C=S stretching mode of TU) is significantly lowered (gray line, Fig. 2c), but it remains unaffected by the addition of Zn and Sn salts to the ink (Supplementary Fig. S2 and Fig. 2d). The C=S stretch is not present for $\hbox {TU/Cu}\le 4$, as was also observed by Uhl and coworkers^26^, whereas the other TU modes in the form of N–C–N, C–N, and $\hbox {NH}_{2}$ are unaffected by Cu-addition. The absence of a Raman band associated with the C=S mode in TU, therefore, suggests that the C=S bond is broken for $\hbox {TU/Cu} \le 4$, and that four TU molecules will coordinate one Cu cation with the sulfur atom. Meanwhile, Zn and Sn mixtures appear to have intact C=S bonds even at low TU concentrations, suggesting that they are coordinated by TU to a lower degree (see Supplementary Fig. S2 online).

Moreover, we observe that the C=S stretching mode of TU shifts from 749 to $745\,\hbox {cm}^{-1}$ with increasing TU concentration (Fig. 2d). This effect is more pronounced for Sn, but also seen to some degree for Cu and Zn. Uhl et al. considers this shift an indication that both DMSO and TU compete in the first coordination shell^28^.

Next, we investigate the potential of using Raman spectroscopy to get new insights into the formation of DMSO complexes upon addition of Cu, Zn, and Sn salts. For this purpose, the S=O stretching mode is used as a reference^29^. In this case, a metal ion can bond to the oxygen atom, which lowers the bond order of the S=O bond and decreases the Raman signal. Alternatively, a metal ion can bond to the sulfur atom, which increases the S=O bond order and increases the signal^29^. However, no such variations have been seen in our data. If instead considering the DMSO C–S stretching mode, we do observe a shift from 699 to $703\,\hbox {cm}^{-1}$ with an increasing amount of TU (Fig. 2d). This shift could be caused by the formation of a metal-to-sulfur bond with DMSO. This effect is more pronounced for Cu and Sn than for Zn, but visible for all metals. Zn–S stretching vibrations should be located at 250 and $274\,\hbox {cm}^{-1}$, but are missing, which emphasizes the lack of Zn–S bonds^25^.

In the inks investigated here, the ligands could be thiourea, DMSO, chloride, or water residues from the salts or the atmosphere, or a mixture of all. Our findings indicate that the Cu complexes consist of metal-to-sulfur bonds with four thiourea molecules (lack of C=S stretching mode of TU), but we also see indications of metal-to-sulfur bonds with DMSO (shift of C–S antisymmetric stretching mode of DMSO). Meanwhile, Zn and Sn are not as notably coordinated by sulfur in TU, but the complexes consist of both TU and DMSO (shift of C=S stretching mode of TU). The Sn complexes, in particular, seem to form metal-to-sulfur bonds with DMSO (shift of C–S antisymmetric mode of DMSO). In this study, a part of the complexes have been mapped out, and it is very likely that a mixture of several complexation mechanisms occupy the inner shells of the metal cations, and that nitrogen in TU, water, or Cl also partake in the coordination.

#### The effect of filtering and mixing time

Once the salts have been weighed and combined with the solvent, they will start to dissolve. In this section, we investigate whether mixing time or filtering can affect the dissolution or complexion mechanism. This analysis is carried out by measuring the composition of the ink after preparation. Three inks with similar cation concentrations (named Ink # 1 to # 3 in Table 3) were studied by inductively coupled plasma optical emission spectroscopy (ICP-OES), and the ratio between individual cations and the total metal content was compared. The optimized precursor compositions had Cu/Me, Zn/Me, and Sn/Me ratios of 0.35, 0.35, and 0.29, respectively, which correspond to a Cu:Zn:Sn ratio of 1.2:1.2:1. This is far from the 1.9:1.2:1 Cu:Zn:Sn composition we expect in highly-efficient CZTS devices^1^, and especially the amount of Cu is low. This is not surprising taking into account that Sn is volatile and that $\hbox {ZnCl}_2$ is hygroscopic, i.e. it readily absorbs water thus likely lowering the true Zn content. Furthermore, other phenomena could affect the composition of final absorber films, such as selective re-dissolution of certain phases or complexes during spin-coating.

**Table 3:** Overview of ink samples and measured ICP-OES cation concentrations. *F denotes filtered and *NF* no filter.

Ink	TU/Me	Mixing time (h)	Filtering*	Cu/Me	Zn/Me	Sn/Me
Added salts				0.35	0.35	0.29
# 1	4	24	F	0.335	0.467	0.198
# 2	4	2	F	0.335	0.471	0.194
		24	F	0.337	0.469	0.194
			NF	0.334	0.471	0.195
		48	F	0.336	0.470	0.194
# 3	2	24	F	0.334	0.470	0.195
			NF	0.333	0.472	0.195

**Figure 3:**
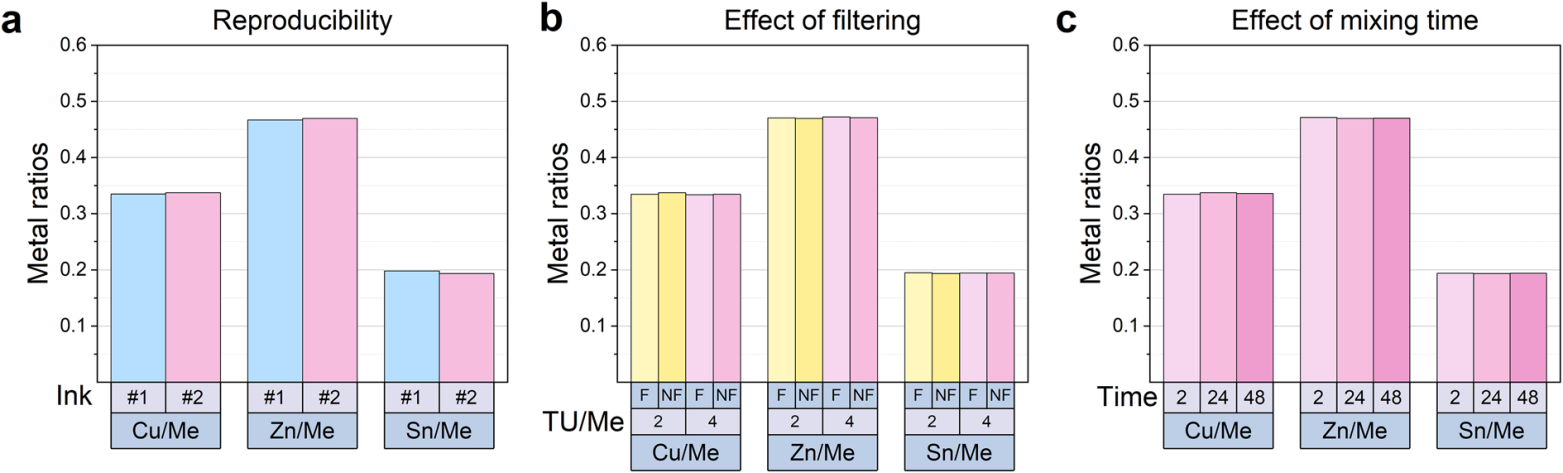
Metal ratios of added salts and determined by ICP-OES on ink samples. Investigation of (**a**) reproducibility, (**b**) effect of filtering, and (**c**) effect of mixing time. Different colors correspond to different inks, where Ink # 1 is blue, # 2 pink, and # 3 yellow. In (**b**), *F* denotes filtered and *NF* no filter.

Firstly, we assess the reproducibility of the ink formulation itself by comparing the composition of two inks prepared similarly, Inks # 1 and # 2, after 24 h mixing time, filtered (Fig. 3a and Table 3). As seen in the data, the metal ratios differ by 0.002, 0.003, and 0.005 for Cu/Me, Zn/Me, and Sn/Me, respectively, or by 0.6%, 0.6%, and 2.3% relative to the lowest number. These values lie within the $\pm 3\%$ accuracy of the measurement itself, and the experimental error of the weighing procedure cannot be further assessed with this method.

Secondly, we assess the effect of filtering the ink. Fig. 3b shows the composition of two inks with different TU/Me ratios, namely Inks # 2 and # 3, after 24 h of mixing time, with and without filter. Any weighing error can be disregarded when comparing measurements made on the same ink, i.e. in this case the filter (*F*) vs. no filter (*NF*) measurements. Very small changes are observed in the cation concentrations with and without filtering, with the largest differences of 0.9% for Ink # 2 and 0.3% for Ink # 3.

Mixing time was expected to affect the cation concentration, as the ink appeared blurry after only 2 h of mixing (not shown). The weighing error can also be disregarded for this study, as all samples originated from the same mother solution. Figure 3c reveals that all cation ratios are similar, and the investigated mixing times do not affect the cation concentrations.

In conclusion, when working with several milliliters of sample, the composition is on average not affected by neither filtering nor the mixing times investigated here. The reproducibility could only be assessed within the $\pm 3\%$ accuracy of the measurement itself, but nonetheless to improve the weighing procedure, we suggest exchanging the hygroscopic salt, as well as working with larger amounts to minimize variations.

#### Thermal behaviour of the ink

**Figure 4:**
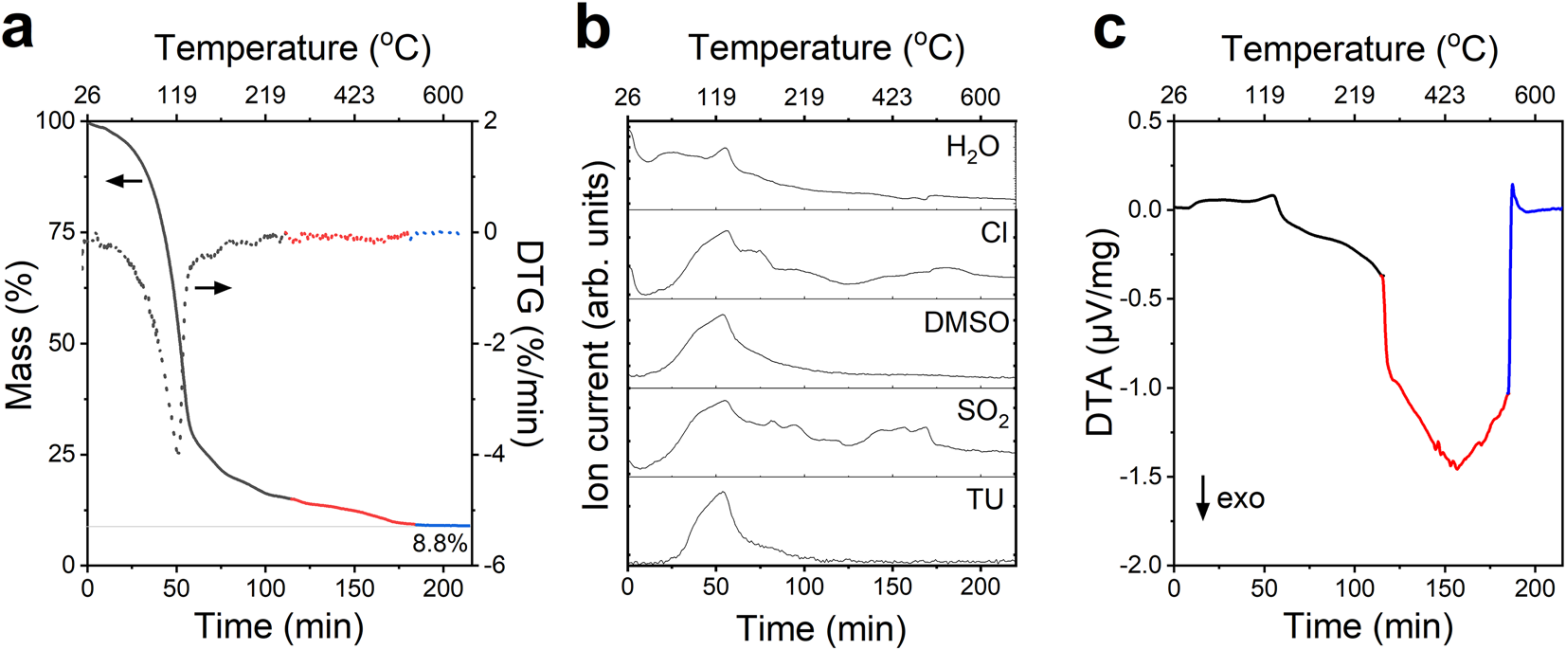
Investigation of the thermal behaviour of the ink. (**a**) TGA and DTG signal for an aprotic molecular ink sample in air. (**b**) Selected mass-to-charge ratios as a function of time and temperature. The signals for $\hbox {H}_{2}\hbox {O}$ (*m/z* 18), Cl (*m/z* 35), DMSO (*m/z* 63), $\hbox {SO}_2$ (*m/z* 64) and TU (*m/z* 76) were characteristic for all detected gases. (**c**) DTA measurement, where the term “exo” denotes an exothermic reaction. Three different heating ramps were used, which is illustrated by the different coloured lines in (**a**, **c**). The black curve was heated at 2 K/min (20 to $250\,^{\circ }\hbox {C}$), the red at 5 K/min to $600\,^{\circ }\hbox {C}$, and the blue curve is an isothermal step at $600\,^{\circ }\hbox {C}$.

Thermogravimetric analysis (TGA) and differential thermal analysis (DTA) were carried out to study what happens inside the ink as the temperature rises and was used to determine interesting pre-annealing conditions. The mass loss in air is shown in Fig. 4a together with the differential thermogravimetric (DTG) curve, i.e. the first derivative of the mass with respect to time. The data indicate that the main mass loss event occurs at $126\,^{\circ }\hbox {C}$ and is initiated with a steep mass loss starting at $103\,^{\circ }\hbox {C}$. The mass spectrometry (MS) data (see Fig. 4b) reveals that all ink constituents break down or evaporate, i.e. water ($T_{\mathrm {boil}}=100\,^{\circ }\hbox {C}$), DMSO ($T_{\mathrm {boil}}=189\,^{\circ }\hbox {C}$), thiourea ($T_{\mathrm {melt}}=176\text {-}178\,^{\circ }\hbox {C}$, $T_{\mathrm {sublime}}=150\text {-}160\,^{\circ }\hbox {C}$), and Cl. The mass spectrum signals for thiourea and DMSO peaks at $125\,^{\circ }\hbox {C}$, while water and chloride peaks at $130\,^{\circ }\hbox {C}$.

A continuous removal of solvent, additives, or loss of volatile elements are seen as several smaller peaks in the DTG curve until the isothermal step at $600\,^{\circ }\hbox {C}$ is reached after 185 min. In particular, the MS data shows that $\hbox {SO}_2$ and Cl are again released between 325 and $600\,^{\circ }\hbox {C}$ (Fig. 4b), and accounting for the mass loss between 250 and $600\,^{\circ }\hbox {C}$ (Fig. 4a). $\hbox {SO}_2$ does not exist in the ink, but it could form when DMSO is oxidized. During the isothermal step at $600\,^{\circ }\hbox {C}$, the mass loss of the sample basically becomes zero. The differential thermal analysis (DTA) measurement in Fig. 4c indicates an initial endothermic reaction, e.g. thermal decomposition of organic material, followed by an exothermic reaction, such as oxidation. A faster heating rate reduces the resolution and increases the DTA signal, as seen in the data.

The calculated initial mass and expected mass loss, as well as the measured final mass loss, are shown in Table 4, where the expected mass loss is derived from the composition measured by energy dispersive X-ray spectroscopy (EDX). The sample consisted of approximately 92% of solvents and additives, and since 8.8% of the original sample remains, it suggests that most residues are removed. If we look at the expected mass loss in more detail, we can calculate the expected total mass loss of 92.9% (see Table 4). This is obtained by assuming that all additives are removed, that there is no loss of Cu and Zn, that we end up with Cu/(Zn+Sn) = 0.76 (determined as an average of several annealed film by EDX), and that there are four S atoms per Sn atom. When comparing this value to the measured mass loss, which was 91.2%, we find that 1.7% of the additives have not been removed from the sample. Some errors to this estimation include that (1) the solvents could evaporate before the measurement started, or (2) the sample might oxidize when heated to $600\,^{\circ }\hbox {C}$. No characterization was carried out on the product, however, the formation of oxide or sulfoxide species would change the weight of the sample^30^, due to their differences in masses, oxygen (*Z* = 8) and sulfur ($\textit{Z}=16$).

**Table 4:** Calculated initial mass percentages and expected mass loss, as well as the measured final mass.

	Calculated		Measured
	Initial mass (%)	Mass loss (%)	Mass loss (%)
DMSO	74.3	74.3	–
TU	9.5	8.0	–
Water	2.1	2.1	–
Cl	6.6	6.6	–
Cu	2.2	0	–
Zn	2.2	0	–
Sn	3.1	1.9	–
Sum	100	92.9	91.2
Remaining mass	–	7.1	8.8

The initial mass is determined from the added salts and the expected mass loss from the composition measured by EDX.

Typical pre-annealing temperatures in literature for spin-coated CZTS and CZTSSe range from 350 to $540\,^{\circ }\hbox {C}$ when done in nitrogen, however for air, temperatures between 300 and $350\,^{\circ }\hbox {C}$ are used (see Table 1). Based on this study and the literature on the topic, we chose pre-annealing temperatures of $250\,^{\circ }\hbox {C}$, $350\,^{\circ }\hbox {C}$, and $450\,^{\circ }\hbox {C}$ for our further investigations, as they would allow removal of 93%, 95% and 97% of the ink residues, respectively.

### Thermal exposure during pre-annealing

We then investigate the effect of thermal exposure during pre-annealing. Thin films were spin-coated and prepared with increasing thermal exposure (time and/or temperature) during pre-annealing. The pre-annealing treatment was carried out for each spin-coated layer until the desired thickness was achieved, which added up to ten times for each sample. The following samples were prepared: $250\,^{\circ }\hbox {C}$ for 10 s, 30 s, and 60 s, $350\,^{\circ }\hbox {C}$ for 30 s, and $450\,^{\circ }\hbox {C}$ for 30 s with and without filter. All samples were annealed in the presence of sulfur at $595\,^{\circ }\hbox {C}$ for 30 min.

#### Film morphology

**Figure 5:**
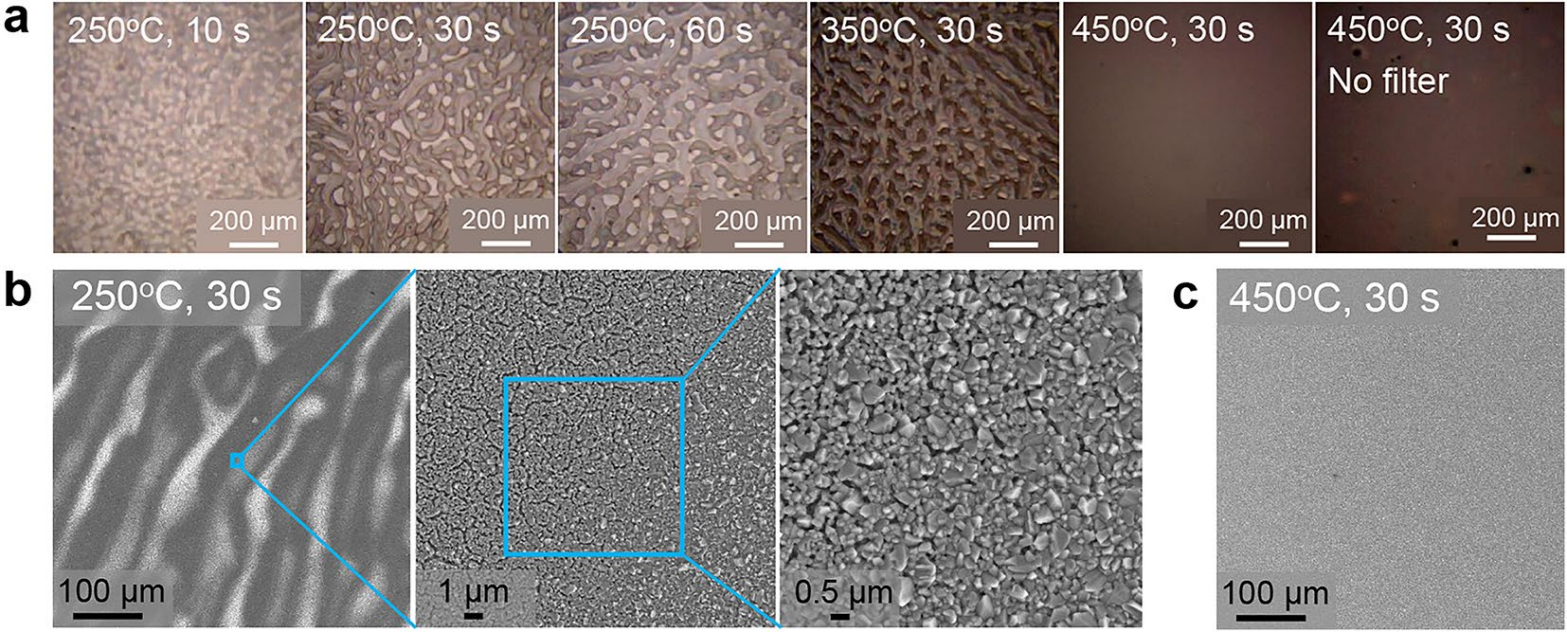
Film morphology as a function of increasing thermal exposure during pre-annealing. (**a**) Optical microscopy images of annealed films for increasing pre-annealing temperatures, with and without filter. (**b**) SEM images depicting the film inhomogeneities in the so-called worm-like pattern of a sample pre-annealed at $250\,^{\circ }\hbox {C}$ for 30 s for increasing magnification. The blue boxes show the magnified areas. (**c**) SEM image of a sample pre-annealed at $450\,^{\circ }\hbox {C}$ for 30 s.

A worm-like pattern takes shape in the deposited film, as observed in the optical microscope images of the films pre-annealed at $250\,^{\circ }\hbox {C}$ and $350\,^{\circ }\hbox {C}$ (see Fig. 5a). The worm-like pattern stems from hydrodynamic processes taking place within the drying film at temperatures below $450\,^{\circ }\hbox {C}$. A phenomenon similar to Rayleigh–Bénard convection is responsible for this patterning: This pattern forms when a temperature gradient between the upper and lower interfaces of a liquid film establishes convective rolls when a critical Rayleigh number is surpassed. Consequently, a pattern emerges based on buoyancy variations and this effect has been used to describe the worm-like pattern of e.g. nanoparticle inks^31,32^.

The worm-like pattern results in increased surface roughness and—on a micro-scale—structural inhomogeneities in the form of differences in film density. This is visible on the SEM images in Fig. 5b, where an area containing one high and one low contrast material is imaged. Cracks are present in some areas, resulting in a varying porosity across the film. This roughness could potentially also cause secondary phases or compositional variations. Increasing the pre-annealing temperature to $450\,^{\circ }\hbox {C}$ prevents the pattern-formation and forms a flat, uniform film (Fig. 5c). This finding could describe why such a high deposition temperature is currently used for several of these systems^5,7,14,19^.

**Figure 6:**
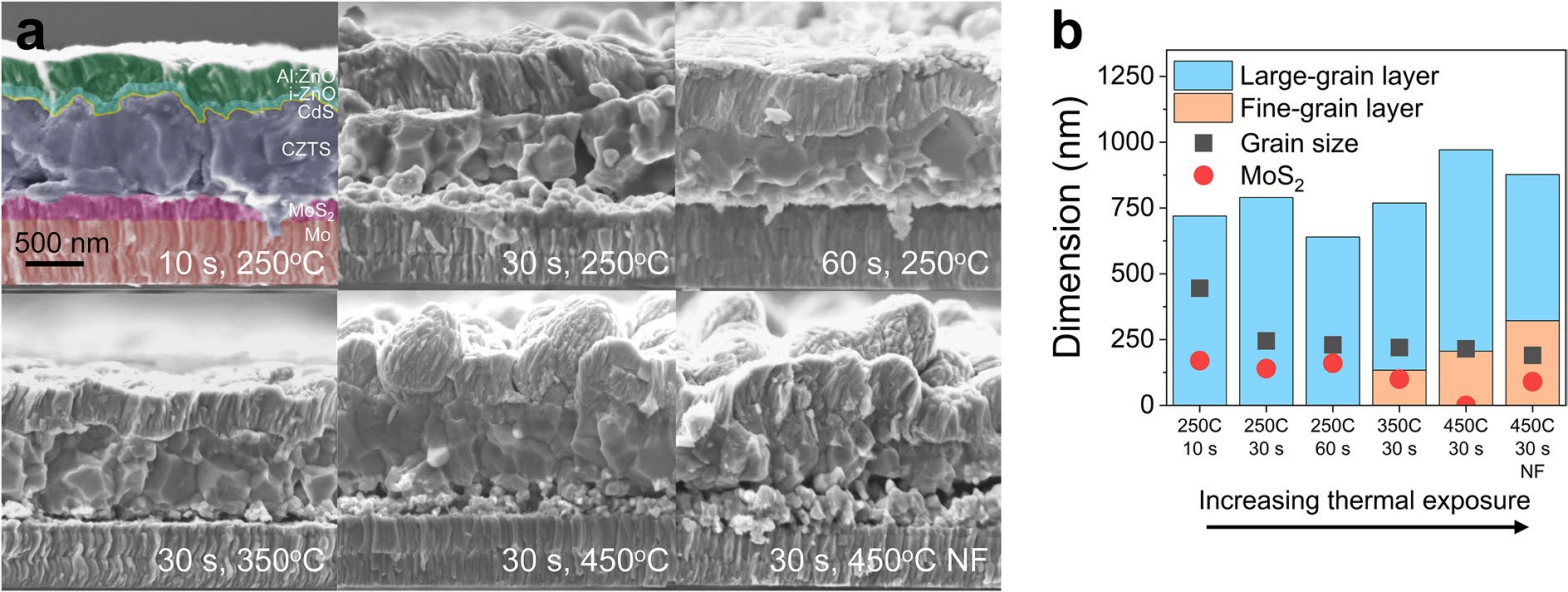
Film microstructure as a function of increasing thermal exposure during pre-annealing. (**a**) Cross-section SEM images of CZTS solar cells prepared under various pre-annealing treatments. (**b**) Diagram summarizing the dimensions extracted from the SEM images in (**a**).

To further investigate the microstructure of the films, SEM images of the cross-sections were obtained. These are shown in Fig. 6a and selected extracted dimensions are summarized in the diagram in Fig. 6b. As temperature increases, a fine-grain layer is created at the CZTS/Mo interface. Grain sizes were determined in the software ImageJ from at least 100 grains on the top view SEM images such as the ones in Supplementary Fig. S3. Large grains are formed, especially at low thermal exposure considering these films are made without additional Na or other sintering agents. Furthermore, a relatively thick $\hbox {MoS}_2$ layer is created at lower thermal exposure, again suggesting a more complete sulfurization. $\hbox {MoS}_2$ is commonly seen at the interface between Mo and the kesterite, and the electrical properties of $\hbox {MoS}_2$ vary depending on orientation and indiffusion of elements from the device stack^33^. Hence, it is difficult to evaluate the effect of the $\hbox {MoS}_2$ layer in our devices, however the fact that it forms is a sign that the sulfurization has been effectively reaching the back of the CZTS layer.

#### Phase characterization

**Figure 7:**
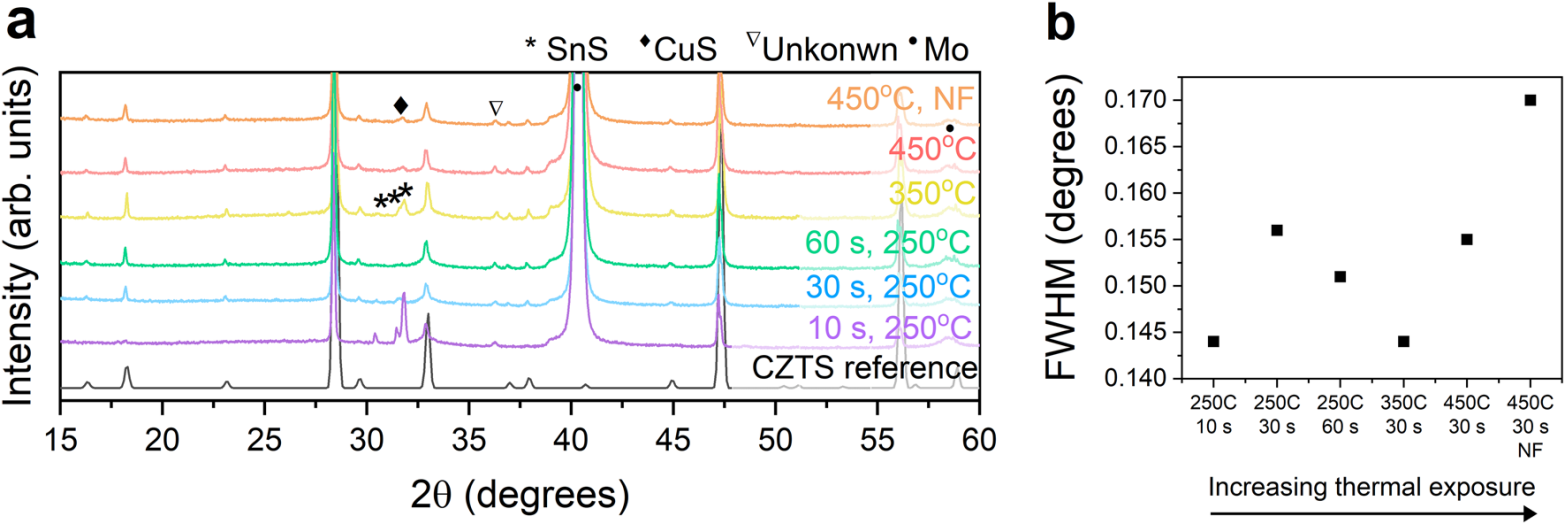
XRD analysis of annealed films. (**a**) XRD patterns of annealed films prepared under different pre-annealing conditions. (**b**) FWHM of the main CZTS (112) peak.

The XRD patterns of annealed films prepared under different pre-annealing conditions are displayed in Fig. 7a along with the reference CZTS pattern (ICSD coll. code 171983). All CZTS peaks are detected in all samples, except for the sample pre-annealed at $250\,^{\circ }\hbox {C}$ for 10 s. The corresponding Raman spectra of the $250\,^{\circ }\hbox {C}$, 10 s sample (see Supplementary Fig. S4 online) indicates that the material is not CZTS but rather cubic or monoclinic $\hbox {Cu}_{2}\hbox {SnS}_{3}$. This stresses the importance to carry out Raman spectroscopy, as XRD cannot distinguish between tetragonal CZTS, tetragonal $\hbox {Cu}_{2}\hbox {SnS}_{3}$, tetragonal $\hbox {Cu}_{3}\hbox {SnS}_{3.6}$, and cubic ZnS (ICSD coll. codes 50965, 237555, and 77090, respectively). The particular sample prepared at $250\,^{\circ }\hbox {C}$ for 10 s furthermore contains a large amount of orthorhombic SnS (ICSD coll. code 24376) after annealing according to the XRD pattern. In addition, it has one of the lowest FWHM of the “kesterite” (112) peak among all samples (see Fig. 7b), which is in agreement with the average grain size observed in SEM (see Fig. 6).

The remaining samples display the XRD peaks characteristic for CZTS, and it is therefore likely that the absorbers contain CZTS, however we cannot exclude the existence of Cu–Sn–S and ZnS phases. Smaller amounts or potentially less crystalline orthorhombic Sn–S is to some degree also present in all other samples, and could exist in a range of different phases (SnS (space group *Pnma*), SnS (space group *Cmcm*), or $\hbox {Sn(SnS}_{3}$) with ICSD coll. codes 24376, 52106, and 15338, respectively). Nonetheless, this secondary phase does not necessarily affect device performance, as the etch carried out before CdS-deposition removes Sn-S phases^34,35^. Certain peaks could not be identified, i.e. the one at $2\theta =36.3^{\circ }$, however it has been observed before in CZTS and $\hbox {Cu}_{2}\hbox {SnS}_{3}$ films^36^. Several other phases could potentially exist, such as hexagonal CuS (ICSD coll. code 32105), but it could not be definitively identified due to a lack of supportive peaks. The detection limit of standard laboratory XRD is lower than synchrotron sources, and even though a material appears phase-pure in laboratory XRD, potentially it is not^37^.

**Figure 8:**
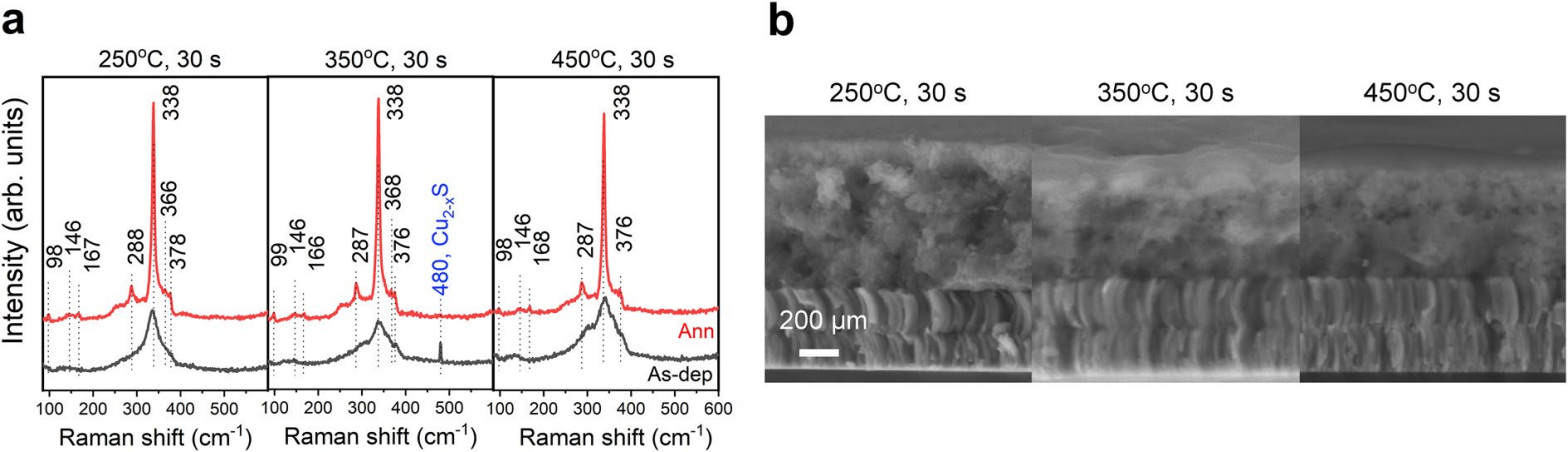
Raman analysis of as-deposited and annealed films. (**a**) Raman spectra of as-deposited and annealed samples prepared with pre-annealing conditions of $250\,^{\circ }\hbox {C}$, $350\,^{\circ }\hbox {C}$, and $450\,^{\circ }\hbox {C}$ for 30 s. The characteristic CZTS peaks are marked, as well as the position of the $\hbox {Cu}_{2-x}\hbox {S}$ phase. (**b**) Cross-section SEM images of as-deposited films.

Raman spectroscopy was carried out on selected as-deposited and annealed films, and the spectra are shown in Fig. 8a. The annealed films display the characteristic CZTS peaks^38–40^. No significant difference in morphology could be detected between the as-deposited samples (see Fig. 8b), as they appear equally amorphous and display an indication of a layered structure. All as-deposited samples show a broad Raman peak centered at $338\,\hbox {cm}^{-1}$. This is the main peak in tetragonal CZTS^38–40^, however it is also the most pronounced one in tetragonal $\hbox {Cu}_{2}\hbox {SnS}_{3}$^41,42^. As no other peaks are observed, it is hard to determine which material has formed. Due to the broad nature of the peak, a range of Cu–Sn–S phases could be present, including orthorombic $\hbox {Cu}_{3}\hbox {SnS}_{4}$, as well as monoclinic and cubic $\hbox {Cu}_{2}\hbox {SnS}_{3}$^41,42^. In addition, the sample prepared at $350\,^{\circ }\hbox {C}$ for 30 s displays a Raman shift of 480 cm$^{-1}$, corresponding to $\hbox {Cu}_{2-x}\hbox {S}$, in several locations on the film^41^. The $\hbox {Cu}_{2-x}\hbox {S}$ phase typically forms at $350\,^{\circ }\hbox {C}$^43^ and under Cu-rich conditions, this phase has been shown to result in larger grains^44^. This is consistent with the low FWHM (see Fig. 7b) and large grains in the cross-section SEM image (Fig. 6a) of the sample prepared at $350\,^{\circ }\hbox {C}$.

The average composition of annealed films measured by EDX is shown in Supplementary Fig. S5. As the films are less than $1\,\upmu \hbox {m}$ thick, and as secondary phases are present in the samples, large variations are seen in the measurements. A loss of Sn and perhaps Zn is seen compared to both the ICP-OES ink measurement and added salt concentration, which is expected in order to achieve the desired composition. The compositional variations can also be related to secondary phase segregation and out-diffusion of elements to the front and back surfaces, which would cause changes in the EDX signal.

#### Device performance

**Figure 9:**
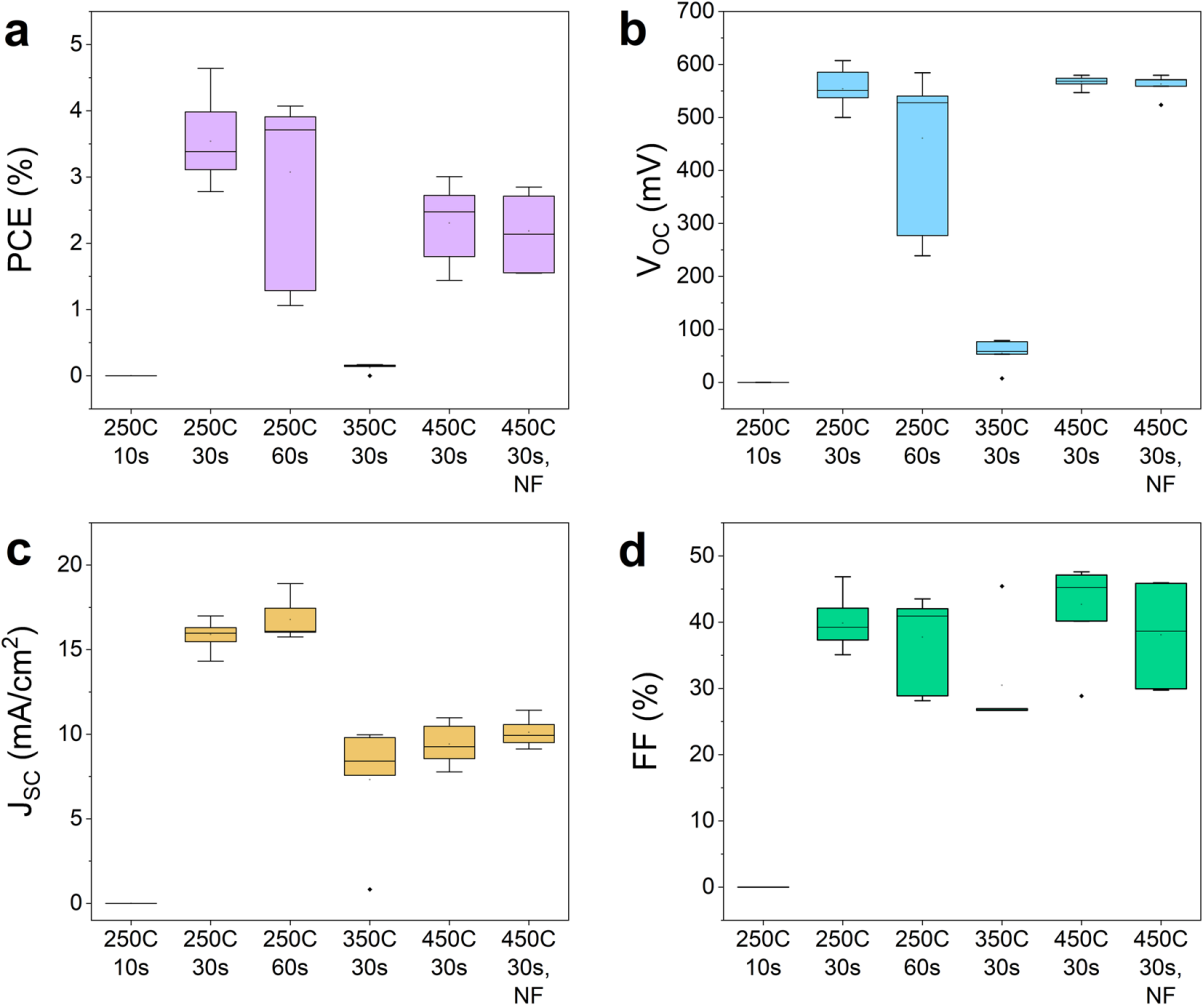
Box plots illustrating device characteristics of samples with different pre-annealing treatment. Including PCE (**a**), $V_{\mathrm {OC}}$ (**b**), $J_{\mathrm {SC}}$ (**c**), and *FF* (**d**).

**Figure 10:**
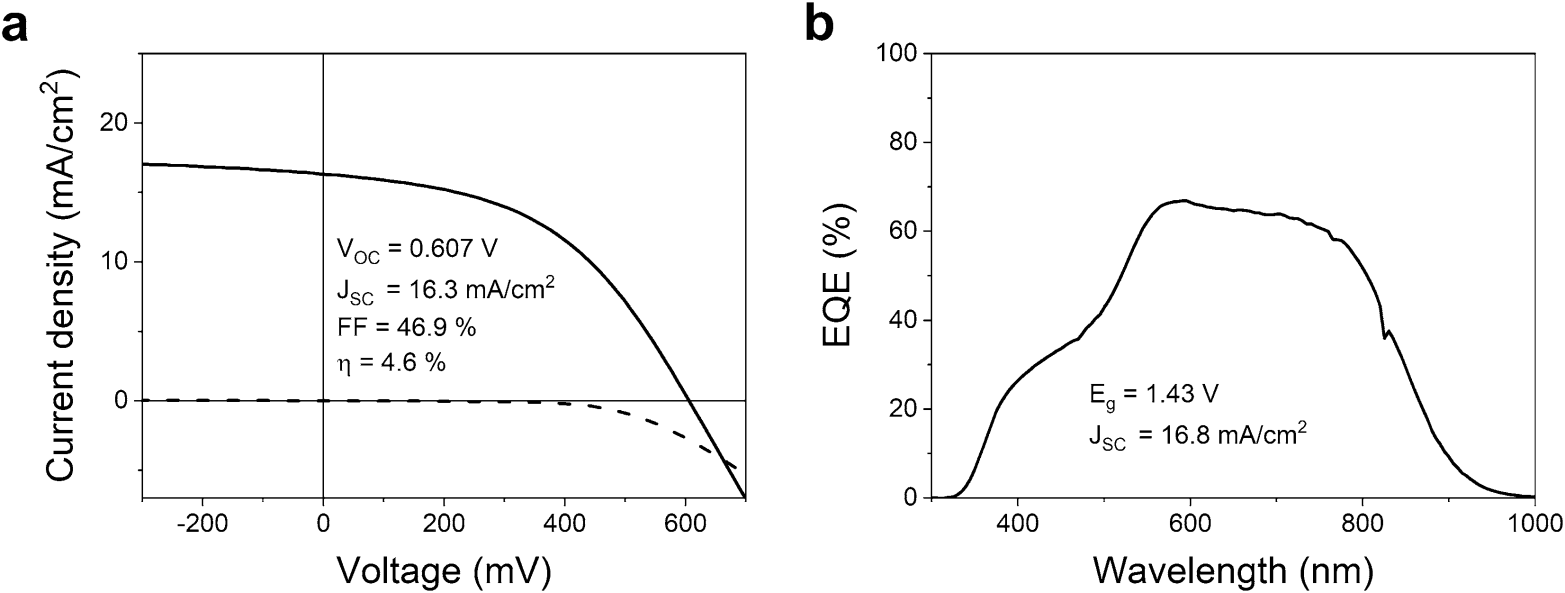
The *J*–*V* curve (**a**) and EQE spectrum (**b**) of the champion device made under pre-annealing conditions of $250\,^{\circ }\hbox {C}$ for 30 s.

Solar cell devices were completed to evaluate the effect of different thermal exposure treatments during pre-annealing on device performance, and box plots summarizing the results are shown in Fig. 9. The current-density-voltage (*J*–*V*) curves of the best cell in each sample are displayed in Supplementary Fig. S6 and the best values listed in Table S1 together with the shunt and series resistances determined in the light. The highest efficiency was achieved under pre-annealing conditions of $250\,^{\circ }\hbox {C}$ for 30 s, and the *J*–*V* curve and external quantum efficiency (EQE) spectrum are displayed in Fig. 10. This treatment resulted in an efficiency of 4.65%, an open-circuit voltage ($V_{\mathrm {OC}}$) of 607 mV, a short-circuit current ($J_{\mathrm {SC}}$) of $16.3\,\hbox {mA/cm}^{2}$, and a fill factor (FF) of 47%. The world record CZTS device fabricated by sputtering had 11%, 730.6 mV, $21.74\,\hbox {mA/cm}^{2}$, and 69.27%, respectively^11^. By comparing these two devices, we see that all parameters are lagging. The EQE spectrum (Fig. 10b) reveals a band gap of 1.43 eV and $J_{\mathrm {SC}}$ of $16.8\,\hbox {mA/cm}^2$. The relatively square profile suggests good collection probability throughout the full absorber layer.

A trend is seen when comparing the efficiency of the devices as a function of thermal exposure. As will be discussed next, we find that the presence of secondary phases is highly detrimental for the device performance. Moreover, a lower thermal exposure is better, as the short-circuit current drops at higher thermal exposure (Fig. 9c). As temperature increases, a fine-grain layer forms at the CZTS-Mo interface and the average grain size becomes smaller, which coincides with the drop in $J_{\mathrm {SC}}$. Moreover, no trend can be seen in the shunt and series resistances (Supplementary Table S1).

Notably, the sample with the lowest thermal exposure ($250\,^{\circ }\hbox {C}$, 10 s) displays 0% efficiency. It consisted of the largest grains, however, some holes were present (see SEM images in Supplementary Fig. S3). As discussed earlier, this material appeared to be a $\hbox {Cu}_{2}\hbox {SnS}_{3}$ phase and not CZTS (see Raman spectra in Supplementary Fig. S4), secondary Sn–S phases were observed (see XRD pattern in Fig. 7a, and the top view SEM image in Supplementary Fig. S3) and a large Zn-loss has occurred (see composition in Supplementary Fig. S5). The loss in Zn seems to be the culprit, and could stem from selective re-dissolution of the film during spin-coating after the very short pre-annealing time. Alternatively, the low Zn concentration could be related to Zn accumulation at the back surface.

At $350\,^{\circ }\hbox {C}$, the efficiency drops to 0%, and this could be related to the Cu–S phases detected in the as-deposited film (see Fig. 8). The Cu–S phases can aid grain growth in the material, however, if not fully incorporated into the CZTS or removed by etching, they will shunt the device. This is backed up by the low shunt resistance of $10\, \Omega \, \mathrm {cm}^{2}$ (Supplementary Table S1) extracted from the corresponding *J*–*V* curve (Supplementary Fig. S6).

The performance of the solar cells prepared at $450\,^{\circ }\hbox {C}$ was only slightly degraded by not filtering the ink. We found that the composition does not depend on filtering and the XRD pattern of the two films does not vary significantly (see Fig. 7). However, the optical microscopy images in Fig. 5a shows that a more uneven film results if the ink is not filtered.

### Proposed formation mechanism

**Figure 11:**
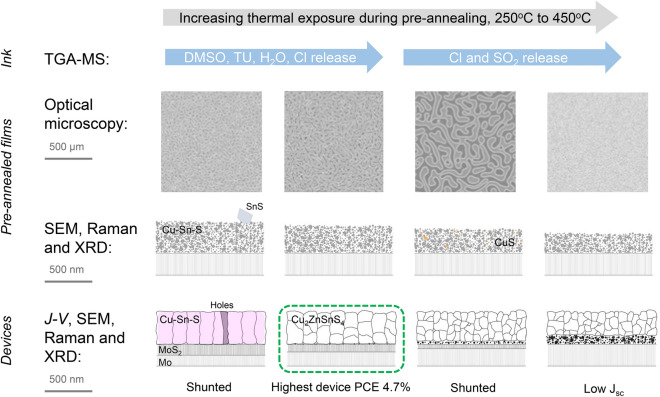
Overview of effects of different thermal exposure during pre-annealing. Figure inspired by^5^.

We have summarized our findings in Fig. 11. As the pre-annealing temperature is increased, the ink constituents start to break down, and on a milimeter-scale, a certain worm-like pattern appears. Above a temperature of $325\,^{\circ }\hbox {C}$, Cl and $\hbox {SO}_2$ species are continually released, and the worm-like pattern is avoided for pre-annealing temperatures above $350\,^{\circ }\hbox {C}$.

In the as-deposited films, a Cu–Sn–S phase was detected in all samples, and SnS flakes and traces of a Cu–S phase in the films prepared at $250\,^{\circ }\hbox {C}$ for 10 s and $350\,^{\circ }\hbox {C}$, respectively.

During sulfurization, several processes take place. First of all, the kesterite CZTS phase has formed in all samples, except the one with the lowest pre-annealing treatment. Additionally, a $\hbox {MoS}_{2}$ layer has formed, but its thickness decreases as the pre-annealing temperature increases, suggesting effective sulfur-diffusion to the back interface at lower heat treatments. This is consistent with the larger grain sizes appearing at lower temperatures. At higher pre-annealing temperatures, a fine-grain layer exists at the back surface, indicating a barrier for a complete sulfurization. The larger grains at lower pre-annealing temperatures might be related to a more amorphous precursor, which is potentially easier to sulfurize, as opposed to the more crystalline Cu–Sn–S films formed at higher pre-annealing temperatures. It is also possible that the lower thermal exposure routes result in more sulfur in the precursor film, which also enhances grain growth. The best working device is the more homogeneous one: without secondary phases and a fine-grain layer.

## Conclusion

In this aprotic molecular ink system, four thiourea molecules coordinate each Cu atom, while a mixture of coordination mechanisms take place for Zn and Sn. Thus, the studies were made with a concentration of thiourea/metal of 4. The mixing times and filtering, which were investigated by ICP-OES, showed no effect on the average composition, however, an uneven morphology was seen when films were deposited without a filter. TGA-MS data indicate that 93% of the solvent was removed at $250\,^{\circ }\hbox {C}$, while chloride and sulfoxide species are released at higher temperatures. An indication of an oxidation reaction is seen as an exothermic peak in the DTA starting at $150\,^{\circ }\hbox {C}$, however, it must be limited as the Raman spectra of as-deposited films showed Cu-Sn-S phases as expected.

Based on the ink investigations, pre-annealing temperatures of $250\,^{\circ }\hbox {C}$, $350\,^{\circ }\hbox {C}$, and $450\,^{\circ }\hbox {C}$ were chosen for further study. At low thermal exposure, a distinct worm-like pattern appears when as-deposited films are inspected in an optical microscope. This pattern disappears and a uniform film appears as temperature is increased. Hence, higher pre-annealing temperatures allow more chloride to disappear and less porosity in the annealed film. Nonetheless, the best devices were made at lower thermal exposure during pre-annealing, because the short-circuit current drops as temperature is increased. The drop in $J_{\mathrm {SC}}$ could stem from the thicker fine-grain layer at the CZTS/Mo interface or the smaller grains.

## Methods

### Ink formulation, deposition, annealing, and device fabrication

Inks are formulated by mixing 852.4 mg copper(II) chloride dihydrate ($\hbox {CuCl}_{2}\times 2\hbox {H}_{2}\hbox {O}$, $\ge 99.95\%$), 681.4 mg zinc chloride ($\hbox {ZnCl}_{2}$, 99.999%), 857.7 mg tin(II) chloride dihydrate ($\hbox {SnCl}_2\times 2\hbox {H}_2\hbox {O}$, $\ge 99.995\%$), and 1408.2 mg thiourea (TU, $\hbox {NH}_{2}\hbox {CSNH}_2$, $\ge 99.0\%$) in 10 ml dimethyl sulfoxide (DMSO, ($\hbox {CH}_{3})_{2}\hbox {SO}, \ge 99.9\%$). The ink was left under stirring for > 1 day and shaken several times to ensure that all salts were fully dissolved, the redox reaction had equilibrated, and the complexation chemistry stabilized.

Films were deposited through spin-coating (on a KW-4A spin-coater from MicroNanoTools) with a chuck suitable for the size of the sample. The substrates used were Mo-coated soda lime glass (SLG) of 1 mm thickness (from ZSW), and they were cleaned in a 1:1 Milli-Q water (from Millipore Corp, $18.2\,\hbox {M}\Omega \,\hbox {cm}$ at $25\,^{\circ }\hbox {C}$) to ethanol ($\hbox {C}_{2}\hbox {H}_{6}\hbox {O}$, 96%) mixture under sonication for 10 min, followed by an ethanol rinse and blow-dried with nitrogen. The ink was filtered through a $0.1\,\upmu \hbox {m}$ nylon filter media with polypropylene housing (from Whatman) and dropped on the substrate for an almost complete coverage. The spin conditions used consisted of a two-ramp program, first with 3 s at 750 rpm to evenly distribute the ink on the substrate followed by 25 s at 2000 rpm to spin off any excess ink and obtain a smooth, uniform layer. The sample was transferred to a hot-plate (C-MAG HP 7 from IKA) set at a specific temperature and left for a certain time in air, whereafter it was cooled for more than 2 min. The process was repeated ten times until the desired thickness was achieved.

The as-deposited samples were sulfurized in a closed graphite box (from Mersen Nordic) containing 100 mg S powder (99.998%) in a tube furnace (from Hobersal). The chamber was evacuated to a base pressure of $<10^{-2}$ mbar, and purged with nitrogen three times to ensure complete removal of oxygen and water. The annealing profile used a ramp rate of $10\,^{\circ }\hbox {C/min}$ to a peak temperature of $595\,^{\circ }\hbox {C}$ and a peak pressure of 250 mbar, where it dwells for 30 min, followed by natural cooling.

Before buffer-layer deposition, the samples were etched in a 10 wt% solution of ammonium sulfide (($\hbox {NH}_4)_2\hbox {S}$) in Milli-Q water. Thereafter, a 60 nm CdS buffer layer was deposited by chemical bath deposition and a 400 nm ZnO/Al:ZnO transparent conductive oxide was deposited by sputtering. Thus, full cells consist of SLG/Mo/CZTS/CdS/ZnO/Al:ZnO, and neither contacts nor anti-reflective coatings are used. All devices were post annealed at $275\,^{\circ }\hbox {C}$ for 1 min in nitrogen atmosphere.

### Characterization

Liquid samples are characterized by Raman spectroscopy, inductively coupled plasma optical emission spectroscopy (ICP-OES), and simultaneous thermogravimetric and differential thermal analysis (STG) coupled with mass spectrometry (MS). Raman spectroscopy was done at room temperature on a Reinshaw inVia Reflex confocal microscope, using a diode-pumped solid-state laser with a wavelength of 532 nm. The liquid sample holder from Reinshaw was used. The laser beam was focused onto the samples with a VIS-NIR 5 $\times$ objective. The measurements were carried out in back-scatter configuration and the spectra were calibrated with the Raman peak of Si at $520.5\,\hbox {cm}^{-1}$.

For the ICP-OES measurements, 0.2–0.3 g sample solution is weighed and transferred quantitatively into a teflon microwave digestion vessel in duplicates. 9 ml 37% HCl (p.a. quality) and 3 ml 65% $\hbox {HNO}_3$ (p.a. quality) is added and the sample is digested at $200\,^{\circ }\hbox {C}$ for 20 min in a microwave digestion unit (from Anton Paar Pro) resulting in a clear sample solution. The sample is then transferred to a 100 ml volumetric flask and filled to the mark with Milli-Q water. The Cu, Zn, Sn, and S content in the samples are quantified by ICP-OES with an Agilent 720 ES ICP-OES instrument. Suitable dilutions are used for the analysis. The emission signals from several Cu, Zn, Sn, and S specific emission lines are compared to the signals from certified calibration standards containing 0–10 mg/l Cu, Zn, Sn, and S. The precision of the analysis is $\pm 3\%$ relative with 95% confidence.

The thermal analysis (STG-MS) was performed on a Netzsch STA 409CD instrument^30^. Liquid samples were placed in a corundum ($\hbox {Al}_2\hbox {O}_3$) crucible with a pierced lid. The measurement was carried out in air and with a flow rate of 50 ml/min, as this resembles the pre-annealing conditions. The temperature profile selected consisted of a heating rate of $2\,^{\circ }\hbox {C/min}$ from 20 to $250\,^{\circ }\hbox {C}$, followed by a heating rate of $5\,^{\circ }\hbox {C/min}$ from 250 to $600\,^{\circ }\hbox {C}$, and finally an isothermal step at $600\,^{\circ }\hbox {C}$ for 30 min. The slow initial heating rate was chosen to obtain a better resolution in the temperature range where the solvent boils. Data analysis was carried out on the Proteus61 software. The mass specta were collected with a Netzsch QMS 403 C Aëolos, with a heated transfer line at $300\,^{\circ }\hbox {C}$. The NIST MS Search 2.0 database was used for peak identification. The MS data were processed by dividing by the total ion current and by removing relevant background gases. We refer to our previous work^30^ for more details on the method.

Films are characterized by optical microscopy, scanning electron microscopy (SEM), energy-dispersive X-ray spectroscopy (EDX), Raman spectroscopy, and X-ray diffraction (XRD). Optical microscopy was carried out on in the Raman system described above, and as were the Raman spectroscopy measurements for solid samples. A VIS-NIR 50 objective was used, and each Raman spectrum represents an integration of 10 s at a laser power of 0.1 mW and a spot size of $2\times 2\,\upmu \hbox {m}^{2}$. SEM images were obtained on a Merlin field emission gun electron microscope from Carl Zeiss at 5 kV acceleration voltage with HE-SE2 detector. The average grain size was determined in ImageJ as an average of at least 100 grains. EDX measurements were obtained at 15 kV electron acceleration voltage with a Bruker Quantax 70 system integrated into a Hitachi TM3000 tabletop SEM. XRD measurements were obtained with a $2\theta$ range of 10°–90° using a Bruker D8 Advance diffractometer with Cu $\hbox {K}\alpha$ radiation ($\lambda =0.15418\,\hbox {nm}$). The $\hbox {K}\alpha _2$-signal and background were subtracted from the XRD patterns with the program EVA.

Finished devices were characterized by current-density/voltage (*J*–*V*) measurements and external quantum efficiency (EQE) measurements. *J*–*V* measurements were done with a Newport Sol2A Class ABA steady-state solar simulator under standard test conditions, calibrated with a Si reference cell. EQE measurements were done on a QEXL system (from PV measurements), without bias light or voltage. The setup was calibrated with a Si photodiode (from NIST).

## Supplementary information

Supplementary Informations.
