# Supplementary material for: Spin-coated Cu2ZnSnS4 solar cells: A study on the transformation from ink to film
Source: Sci Rep. 2020 Nov 27;10:20749. doi: 10.1038/s41598-020-77592-z (PMC7699652; doi:10.1038/s41598-020-77592-z)
Supplement: Supplementary file 1 — Supplementary Informations. [file 41598_2020_77592_MOESM1_ESM.pdf]

Supporting information for
Spin-coated Cu_2ZnSnS_4 solar cells: A study on the transformation from ink to film

Sara Engberg[1,*], Filipe Martinho[1], Mungunshagai Gansukh[1], Alexander Protti[1], Rainer Küngas[2], Eugen Stamate[3], Ole Hansen[3], Stela Canulescu[1], and Jørgen Schou[1]

[1]Technical University of Denmark, DTU Fotonik, Roskilde, 4000, Denmark
[2]Haldor Topsøe, Kgs. Lyngby, 2800, Denmark
[3]Technical University of Denmark, DTU Nanolab, Kgs. Lyngby, 2800, Denmark
[*]sleen@fotonik.dtu.dk

ABSTRACT

This is the supporting information for "Spin-coated Cu_2ZnSnS_4 solar cells: A study on the transformation from ink to film". It includes pictures of the ink mixtures and their Raman spectra, top-view SEM images of the annealed absorbers, the Raman spectra of the sample prepared at 250 ◦C for 10 s, EDX measurements of all films, as well as *J*-*V* curves and parameters for the best cell in each sample.

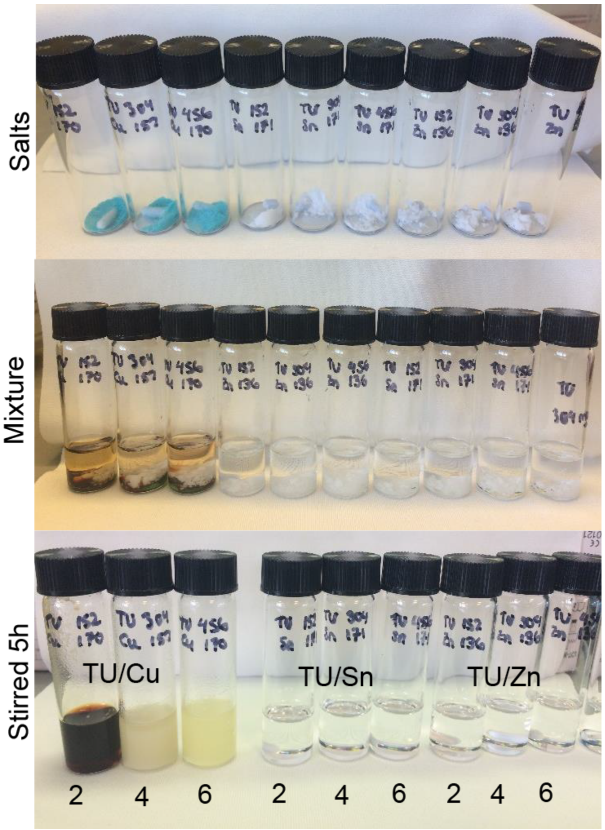

Figure 1. Photographs of vials with salts, mixtures of salts and DMSO, as well as the final mixtures after stirring with Cu, Sn, or Zn at different TU concentrations. The Zn and Sn salts dissolve well in DMSO. The Cu inks are blackish and yellowish, suggesting the presence of Cu(II)S nanoparticles and $Cu(II)Cl_2$ or $Cu(II)DMSO_2Cl_2$, respectively. To obtain the desired Cu(I) oxidation state, a redox reaction is required.

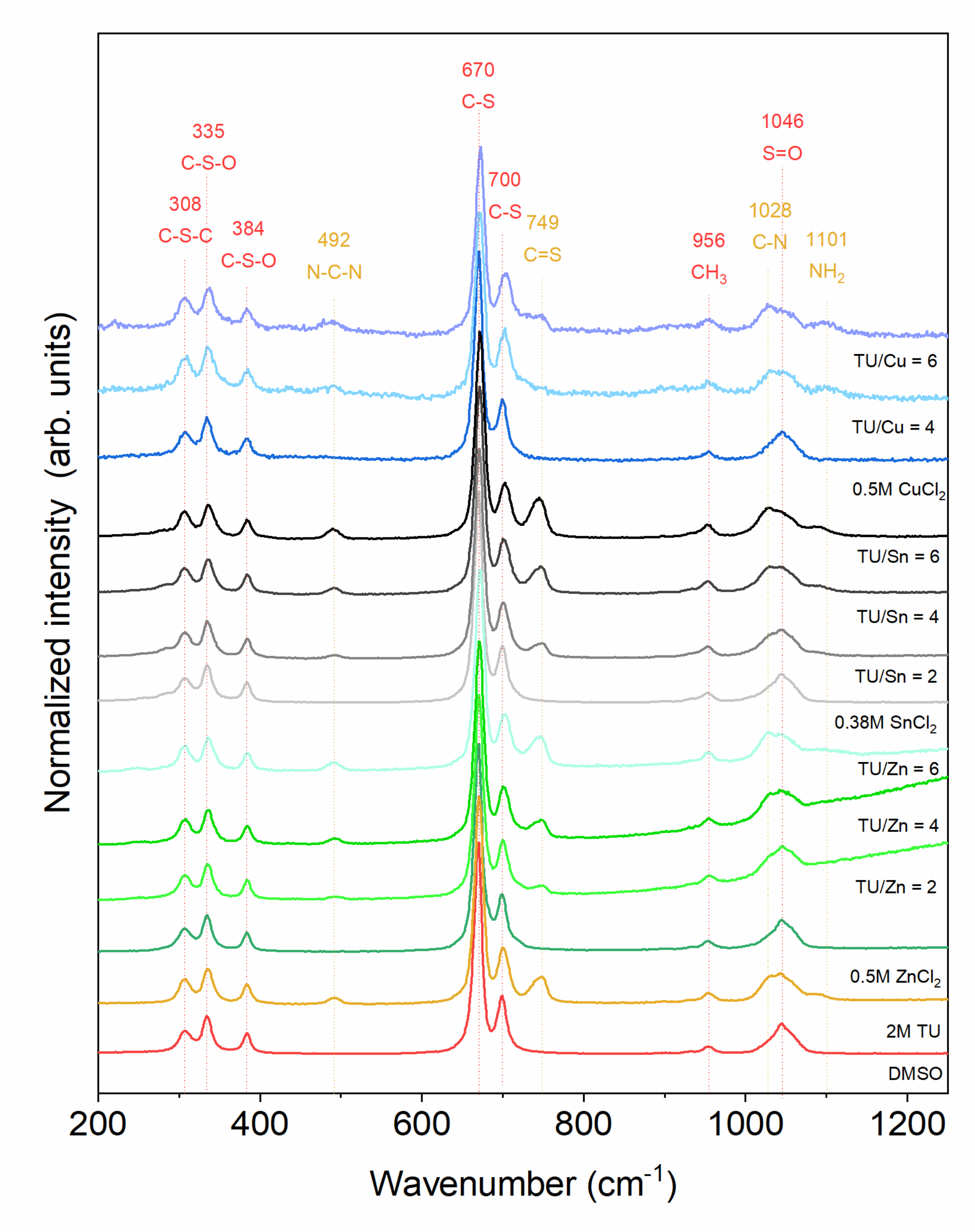

Figure 2. Raman spectra of liquid inks containing DMSO, DMSO and thiourea, Zn and DMSO, Sn and DMSO, Cu and DMSO, as well as mixtures of TU/Cu, TU/Zn, and TU/Sn.

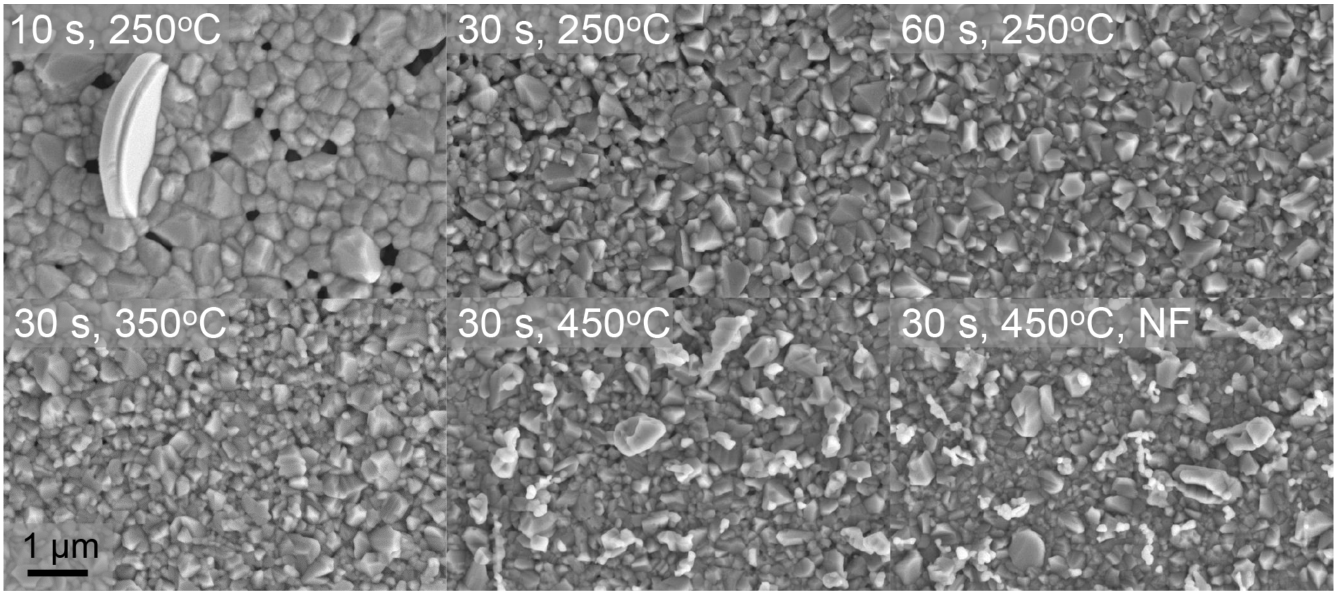

Figure 3. Top view SEM images of annealed films. The sample prepared with the lowest thermal exposure displays large grains, but also SnS flakes and pinholes. EDX measurements revealed that the "flake" contained large amount of Sn.

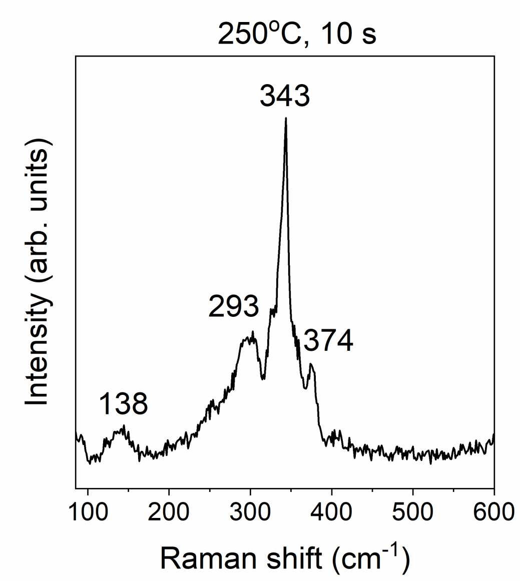

Figure 4. Raman spectrum of sample pre-annealed at 250°C for 10 s. The main 343 cm^{-1} mode suggests that the material is cubic or monoclinic Cu_2SnS_3, but there might be some kesterite mix causing a slight peak shift of the main peak.

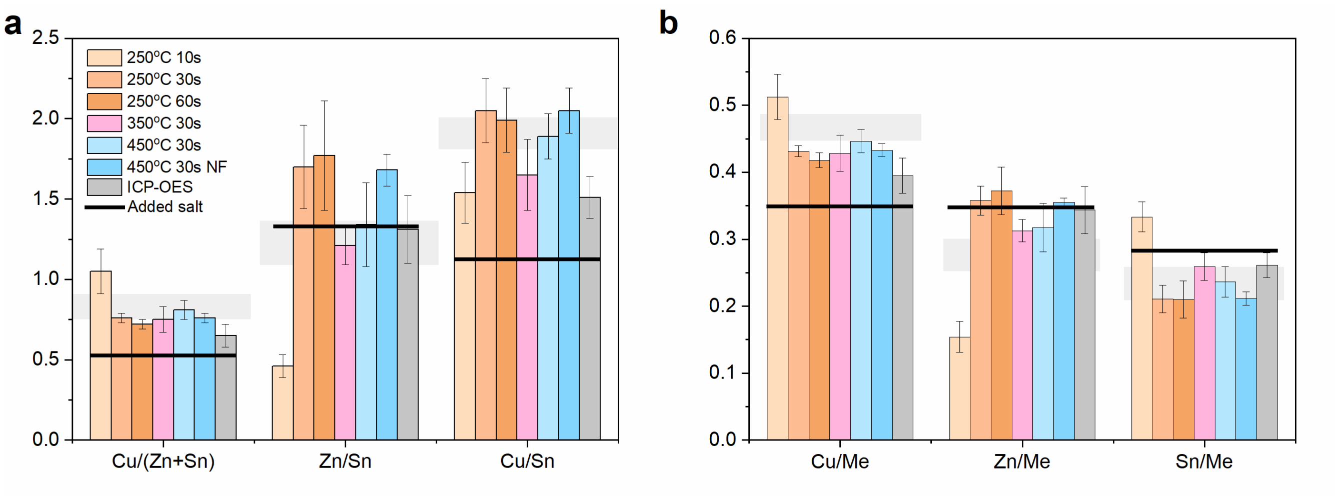

Figure 5. Atomic ratios of annealed films measured by EDX, for (a) relevant CZTS ratios, and (b) metallic ratios. The measured data is compared to the added salt concentration (black line), the desired ratios (grey area), and the ICP-OES ink concentrations. All EDX data are measured on films with TU/Me = 4, made from the same ink, and in all cases a big data spread is seen. The error is possibly due to film thickness variations, but also that SnS phases were seen for the 10 s sample. More Sn and perhaps Zn loss is expected at higher temperatures, and necessary to achieve the desired composition.

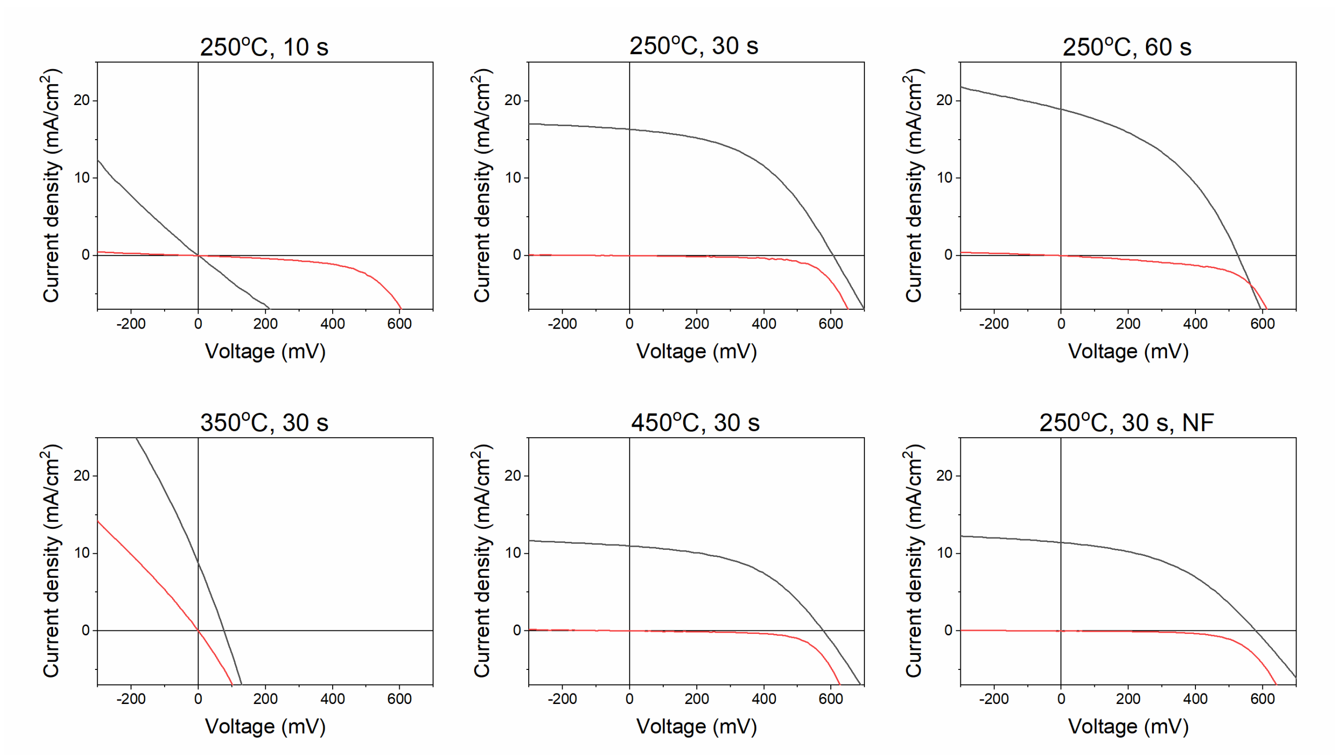

Figure 6. Light and dark *J*-*V* curves of best cells from each sample.

Sample	PCE (%)	V_{OC} (mV)	J_{SC} (mA/cm^2)	FF (%)	R_{SH} (Ω cm^2)	R_S (Ω cm^2)
250°C, 10 s	0	NA	NA	NA	NA	NA
250°C, 30 s	4.64	607	16.3	47	335	7.5
250°C, 60 s	4.07	526	18.9	41	110	5.5
350°C, 30 s	0.17	77	8.8	26	10	4.5
450°C, 30 s	3.00	577	11.0	47	384	6.7
450°C, 30 s, NF	2.85	580	11.4	43	311	5.5

Table 1. Extracted *J-V* curve parameters of best cell from each sample measured in the light. The resistances are determined using Sites' method[1,2].

References

1. Sites, J. R. & Mauk, P. H. Diode quality factor determination for thin-film solar cells. *Sol. Cells* **27**, 411–417, DOI: 10.1016/0379-6787(89)90050-1 (1989).

2. Hegedus, S. S. & Shafarman, W. N. Thin-film solar cells: Device measurements and analysis. *Prog. Photovoltaics: Res. Appl.* **12**, 155–176, DOI: 10.1002/pip.518 (2004).
